# 

**Published:** 1906-06-30

**Authors:** 


### Medical Appointments and Vacancies.

### VACANCIES.

Belfast, Purdysburn Fever Hospital.—Resident Medical Officer. Also House Physician.

Brighton Throat and Ear Hospital, Church Street, Queen's Road.—Non-resident House Surgeon.

Burnley, Victoria Hospital.—Resident Medical Officer.

Chelsea Hospital for Women, Fulham Road, S.W.—Clinical Assistant.

Devonport, Royal Albert Hospital.—Assistant Resident Medical Officer.

Egyptian Government, Ministry of Education.—Professor of Midwifery and Gynæcology. Also Medical Tutor and Registrar to Kasr-el-Ainy Hospital.

Exeter, Royal Devon and Exeter Hospital.—Assistant House Surgeon.

Exminster, Devon County Asylum.—Assistant Medical Officer.

Hospital for Consumption and Diseases of the Chest, Brompton.—Resident House Physicians.

Hull, Royal Infirmary.—Casualty House Surgeon.

Metropolitan Asylums Board Fever and Small-pox Hospitals.—Assistant Medical Officers.

Plymouth, South Devon and East Cornwall Hospital. Assistant House Surgeon.

Queen Charlotte's Lying-in Hospital, Marylebone Road, N.W.—Resident Medical Officer.

Ramsgate General Hospital and the Ramsgate and St. Lawrence Dispensary.—Resident Medical Officer.

Royal Ear Hospital, Dean Street, Soho, London, W. Clinical Assistants.

Ryde, Royal Isle of Wight County Hospital.—Honorary Ophthalmic Surgeon.

St. Marylebone General Dispensary, 77 Welbeck Street, Cavendish Square.—Honorary Surgeon.

Sheffield Royal Hospital.—Honorary Assistant Dental Surgeon.

South Africa, Cape Copper Company, Limited.—Assistant Surgeon.

Stockport Infirmary.—Junior Assistant House Surgeon.

Wandsworth Union Infirmary, St. John's Hill, near Clapham Junction.—Junior Assistant Medical Officer.

Wokingham, Berks, London Open-air Sanatorium, Pinewood. Assistant Medical Officer.

### THE HOSPITAL LIBRARY AND CHARITIES BUREAU.

This institution has been founded as a centre of reference in all matters concerning the establishment and management of charities. It is open to all interested in the subject on payment of a small annual fee, and inquiries by members can be made by post. Write for prospectus to the Librarian, The Hospital Buildings, 28 & 29 Southampton Street, Strand.

Inquiries of the Librarian are answered in this column free of charge. Inquiries to be answered promptly by post must be accompanied by a fee of 2s. 6d

### NOTICE TO CORRESPONDENTS.

**All MSS., Letters, Books for Review, and other matters intended for the Editor should be addressed The Editor, "The Hospital," The Hospital Buildings, 28 & 29 Southampton Street, Strand, W.C.**

**The Editor cannot undertake to return rejected MS., even when accompanied by stamped directed envelope.**

**Notice to Advertisers and Subscribers.**

All Advertisements, Orders for Copies of The Hospital, and Business Communications should be addressed to **THE MANAGER (Not to the Editor),** The Hospital, 28 & 29 Southampton Street, Strand London, W.C.

The Cost of Subscription, post free, is as follows:—Per week, 3½d.; per quarter, 3s. 3d.; per half-year, 6s. 6d.; per annum, 13s. Foreign and Colonial:—Per annum, 19s. 6d., payable, in all cases, in advance.

**NOTICE.—Vol. XXXIX. of "The Hospital" (October 1905 to March 1906), in handsome cloth binding, will shortly be on sale at the office of this Journal, price 10s. 6d. Cases for binding the Numbers contained in this Volume 2s. Index to Vol. XXXIX., 3½d., post free.**

**The Monthly Part for June is now ready. Price 1s., by post, 1s. 3d.**

### NURSING TEXT-BOOKS

A Hall Mark

THE SCIENTIFIC PRESS LTD

PUBLISHERS AND BOOKSELLERS

The Hospital Building

28, SOUTHAMPTON STREET, STRAND. LONDON

of Quality.

The following are a few of our Nursing Books. Space will not permit us to give a full list. We shall, however, be pleased to send a complete Catalogue post free upon receipt of your name and address.

**HOW TO BECOME A NURSE.**
**The Nursing Profession: How and Where to Train.**
Being a Guide to Training for the Calling of a Nurse, with Particulars of Nurse Training Schools in the United Kingdom and Abroad, and an Outline of the Principal Laws affecting Nurses, &c.
Edited by Sir Henry Burdett, K.C.B. **2/-** net, **2/4** post free

**NURSING HINTS TO PROBATIONERS ON PRACTICAL WORK.**
By M. Annesley Voysey. **2/-** net, **2/3** post free

**A HANDBOOK FOR NURSES.**
By J. K. Watson, M.D., M.B., C.M.
New and Revised Edition. Profusely illustrated.
**5/-** net, **5/4** post free

**NURSING: ITS THEORY AND PRACTICE.**
Being a Complete Text-book of Medical, Surgical, and Monthly Nursing.
By Percy G. Lewis, M.D., M.R.C.S., L.S.A.
New Edition, profusely illustrated. **3/6** post free

**NURSING: ITS PRINCIPLES AND PRACTICE.**
By Isabel Adams Hampton.
Third Edition, Revised and Enlarged, illustrated.
**7/6** net, **7/10** post free

**PRACTICAL GUIDE TO SURGICAL BANDAGING AND DRESSINGS.**
By William Johnson Smith, F.R.C.S.
Profusely illustrated (suitable for apron pocket). **2/-** post free

**THE NURSE'S PRONOUNCING DICTIONARY OF MEDICAL TERMS AND NURSING TREATMENT.**
Compiled for the Use of Nurses, by Honnor Morten.
Fifth Edition, Edited and Revised, with Phonetic Pronunciations, by Mary I. Burdett.
Bound in cloth boards, **2/-** post free
In handsome leather, gilt, **2/6** net, **2/8** post free

**THE EXAMINATION OF THE URINE.**
By J. K. Watson, M.D., M.B., C.M., Author of "A Handbook for Nurses."
Demy 16mo, cloth boards. **1/-** net, **1/1** post free

**SURGICAL INSTRUMENTS AND APPLIANCES USED IN OPERATIONS.**
An Illustrated and Classified List, with Explanatory Notes.
By Harold Burrows, M.B. Lond., B.S., F.R.C.S.
**1/6** net, **1/8** post free

**ELEMENTARY ANATOMY AND SURGERY FOR NURSES.**
By William McAdam Eccles, M.S. Lond., M.B., F.R.C.S. L.R.C.P.
Profusely illustrated. **2/6** post free

**ELEMENTARY PHYSIOLOGY FOR NURSES.**

By C. F. Marshall, M.D., B.Sc., F.R.C.S.

Illustrated. **2/-** post free

**SURGICAL WARD-WORK AND NURSING.**
By Alexander Miles, M.D. Edin., C.M., F.R.C.S.E.
Second Edition, Enlarged and entirely Revised. Copiously illustrated. **3/6** net, **3/10** post free

**MEDICAL ELECTRICITY AND LIGHT TREATMENT.**
A Practical Handbook for Nurses.
By Kate Neale, Sister in Charge of the Actino-Therapeutic Department, Guy's Hospital.
Profusely illustrated. **2/6** net, **2/9** post free

**ELEMENTARY TREATISE ON THE LIGHT TREATMENT FOR NURSES.**
By James H. Sequeira, M.D. Lond.
Illustrated. **2/6** net, **2/9** post free

**LECTURES UPON THE NURSING OF INFECTIOUS DISEASES.**
By F. J. Woollacott, M.A., M.D. **2/6** net, **2/9** post free

**SIMPLE SANITATION:**
**The Practical Application of the Laws of Health to Small Dwellings.**
By M. Loane. With Introductory Chapter on Sanitary Legislation, by D. A. Mearns Proser. **1/-** net, **1/2** post free

THE SCIENTIFIC PRESS, LTD.,
**Publishers of Nursing Text-Books, Charts, and Case-Books of all Descriptions,**
28 & 29 SOUTHAMPTON STREET, STRAND, LONDON, W.C.

